# Insights into post-fire establishment of three Alpine conifer species after an experimental fire in Tyrol, Austria

**DOI:** 10.3389/fpls.2026.1771923

**Published:** 2026-03-17

**Authors:** Zhiyi Chen, Markus Neurauter, Moritz Stegner, Ursula Peintner, Ilse Kranner, Andreas Bär, Stefan Mayr

**Affiliations:** 1 Department of Botany, University of Innsbruck, Innsbruck, Austria; 2 Department of Microbiology, University of Innsbruck, Innsbruck, Austria

**Keywords:** Alpine conifers, drought, experimental fire, heat, post-fire stress, seedling establishment

## Abstract

Successful post-fire seedling establishment is critical for restoring the protective function of mountain forests, determining long-term forest regeneration dynamics. However, seedling establishment is a vulnerable phase, and little is known about post-fire dynamics and survival performance of Alpine species. In June 2024, an experimental fire was conducted to assess how fire exposure of seeds and the time of sowing under post-fire conditions influence seedling establishment of three conifer species in Tyrol, Austria. Before the fire, seeds of *Pinus cembra*, *Picea abies*, and *Larix decidua* were sown to assess germination and establishment following fire exposure. After the fire, new seeds of the same species were sown at 10-day intervals over six weeks in the burned and control plots. Next, seedling establishment was monitored, and seedling biomass was determined at the end of September. Air and soil temperatures, as well as edaphic factors, were also monitored during the fire and until the end of September. Under controlled laboratory conditions, fire exposure reduced germination of *P. cembra* by 35.5%, whereas *P. abies* and *L. decidua* were only slightly negatively affected (< 20% reduction). In the burned field, however, only 15.3% of fire-exposed *P. cembra* seeds established seedlings, while no seedlings of *L. decidua* or *P. abies* emerged. *P. cembra* seeds sown immediately after the fire established up to 29.3% (control: 52.0%), whilst *P. abies* and *L. decidua* established only when sown one month after the fire, when soil conditions had improved. *P. abies* reached a maximum establishment of 14.0% and *L. decidua* of 4.7% (control: 23.3 and 7.3%, respectively). After the experimental fire, establishment of the small-seeded species was limited. *P. cembra*, which forms large seeds with ample reserves, established the best from seeds sown directly after the fire. This study provides the first insight into seedling establishment of Alpine conifers and highlights the importance of species selection and sowing timing for post-fire regeneration in mountain forests.

## 1 Introduction

Climate change will increase the frequency and severity of wildfires (Jones et al., 2022; Son et al., 2021), even in boreal and temperate regions (Cunningham et al., 2024; Schunk et al., 2013). Warmer and drier conditions are expected to reduce fuel moisture, thereby increasing plant flammability and creating environments prone to wildfires (Pausas and Paula, 2012; Vacik et al., 2011). Concurrently, intensified human activities, such as increasing demand for recreation and urbanization, have increased ignition risks (Arndt et al., 2013; Ganteaume et al., 2013; Vacik et al., 2011). Wildfires are also increasingly occurring in ecosystems that are not adapted to frequent fire. In these ecosystems, post-fire forest regeneration has emerged as a critical phase of forest recovery.

As in other mountain regions, forests in the European Alps provide important ecosystem services, including protection against natural hazards, conservation of soil and water resources, maintenance of biodiversity, and provision of recreational opportunities (Getzner et al., 2017; Hamilton et al., 2008; Miura et al., 2015; Scheidl et al., 2020). Wildfire risk is particularly relevant in high-elevation Alpine forests, which are dominated by vegetation that is not adapted to fire and is highly sensitive to disturbance (Cerioni et al., 2024; Henner and Kirchengast, 2025; Retana et al., 2012). In particular, conifer-dominated stands are highly flammable and are thus strongly affected by wildfires (Conedera et al., 2018; Getzner et al., 2017; Müller et al., 2015; Vacik et al., 2011). Consequently, the protective function of forests can be damaged or even eliminated. Therefore, maintaining and restoring this protective function depends on successful forest regeneration, which is often impeded by post-fire conditions (Mataix-Solera et al., 2011; Scott et al., 2014).

Post-fire soil conditions are dynamic that can either limit or facilitate forest regeneration. Immediately after a surface fire, dark ashes and partially burned litter typically cover the topsoil, which can strongly alter soil physical and chemical properties (low-intensity fires; Scott et al., 2014; Zhao et al., 2024). Dark ashes enhance the absorption of solar radiation, exposing germinating seeds and establishing seedlings in the near-ground soil to high temperatures (Zhao et al., 2024). During the early post-fire phase, alterations in soil structure may increase porosity and induce water repellency. This reduces water availability and exposes seedlings to drought and salinity stress (Johnstone and Chapin, 2006; Weninger et al., 2019). Furthermore, combustion of organic matter can increase soil ion content (Alcañiz et al., 2016; Granged et al., 2011), pH, and heavy metal concentrations, and induce nutrient imbalances that can further limit plant growth. However, precipitation, ash redistribution, and leaching progressively alter post-fire conditions over time, potentially alleviating some of these initial constraints on seedlings (Certini, 2005; Fernandez-Marcos, 2022; Francos et al., 2019; Granged et al., 2011; Rahimi et al., 2020). Alternatively, leaching of ashes into the soil may improve soil fertility temporarily or permanently (Francos et al., 2019; Outeiro et al., 2008; Scharenbroch et al., 2012), due to their high nutrient content. Thus, post-fire conditions for seedling establishment change substantially over time, and the period of germination and early growth is crucial for seedling establishment success.

Forest regeneration following wildfires depends on successful seedling establishment (Grossnickle and Ivetić, 2022; Nolan et al., 2021; Rodman et al., 2025). Regardless of whether seeds survive fire exposure or are sown after fire, germination and successful establishment must occur under post-fire conditions characterized by heat, drought, and salinity stress (Harvey et al., 2016; McDowell et al., 2008; Meinzer et al., 2011; Whitman et al., 2019). Several studies have examined post-fire seedling establishment in fire-adapted regions, including North America, Mediterranean regions, and Australia (Greene et al., 2007; Lamont et al., 1993; Nolan et al., 2021; Sagra et al., 2018). However, knowledge of Alpine species remains scarce, and available research has largely focused on post-fire regeneration from natural seed banks or natural seed recruitment (Cai et al., 2013; Stewart et al., 2021; Urza and Sibold, 2017). Field-based experiments investigating seedling establishment dynamics over extended post-fire periods remain rare (Grossnickle and Ivetić, 2017), despite their importance for improving reforestation and future forest management strategies.

This study investigated how seedlings of three Central European conifers (*Pinus cembra*, *Picea abies*, and *Larix decidua*) established over the growing season after an experimental fire. Using a controlled field experiment, direct fire exposure of seeds was distinguished from post-fire environmental effects on seeds sown at different times after the fire, thereby capturing temporal variation in seedling establishment. We aimed to provide insights into how exposure of seeds to fire and sowing timing under post-fire conditions influenced seedling establishment. We hypothesized that (i) seedling establishment decreases in fire-exposed seeds and in seeds sown after the experimental fire, reflecting the impact of fire on early establishment, (ii) post-fire seedling establishment varies over time, with the strongest environmental constraints occurring shortly after the experimental fire and progressively weakening over the growing season, and (iii) these post-fire seedling establishment dynamics and performance are species-specific.

## 2 Material and methods

### 2.1 Study area

The study was conducted below the tree line in Praxmar (47° 09’N/11° 06’E, 1670 m a.s.l.), Tyrol, in the Tyrolean Central Alps, Austria (**Supplementary Table 1** and **Supplementary Figure 1**). In April 2023, two 3.8 m² experimental plots were prepared and framed with wooden planks. The two plots were located at the forests edge in a southeast-exposed open area. The nearby forests consisted of *P. cembra* L., *P. abies* (L.) Karst., and *L. decidua* Mill., with an understory comprising *Rhododendron ferrugineum* L., *Vaccinium vitis-idaea* L., and *Vaccinium myrtillus* L. One plot was used for the experimental fire (hereafter, fire plot), and the second served as an unburned control (hereafter, control plot). The upper 30 cm of soil was cleared of stones larger than 10 cm in diameter and covered with a 5-cm layer of litter (small branches, needles, and seed-free old *P. abies* cones) collected from neighboring forests. In May 2024, an additional 5-cm layer of dry litter was added to the fire plot to ensure sufficient fuel load and rapid ignition. The experimental fire took place on 4 June 2024 (day of the year 156, hereafter DOY 156), and subsequent measurements and monitoring continued until 22 September 2024 (DOY 266).

### 2.2 Plant material and experimental design

Seedling establishment was studied in three species in the Pinaceae family: *P. cembra* L., *P. abies* (L.) Karst., and *L. decidua* Mill. *P. abies* seeds were purchased from Landesforstgärten Tirol (Austria), and *P. cembra* and *L. decidua* seeds from Herzog. Baum, Samen und Pflanzen GmbH (Austria). *P. cembra* seeds were stratified by the seed supplier; however, germination tests conducted before the experiment on *P. abies* seeds proved that stratification was not required. Additionally, stratification was not required for *L. decidua* according to ISTA (2024). All seeds were stored in sealed bags at 5 °C until use.

Seedling establishment was analyzed for (i) seeds exposed to the experimental fire and for (ii) seeds sown after the fire. (i) Before the fire (on DOY 155), 50 seeds per species were sown in each of three randomly positioned 0.13 m^2^ sowing units within the fire plot (**Supplementary Figure 2**). (ii) For each post-fire sowing, 50 seeds per species were sown in each of three 0.13 m^2^ sowing units. Post-fire sowing started on the day of the fire (DOY 156) and was repeated every 10 days for six weeks in both fire and control plots (**Supplementary Figure 2**). The three 0.13 m^2^ sowing units selected for each sowing date were distributed randomly across the plots. For both approaches (i) and (ii), seeds were placed 2 cm deep within the intermediate litter layer (roughly 2–5 cm above the soil surface), with minimal disturbance to the ash and litter layers. Seeds were sown in lines to avoid mixing with adjacent seeds. Sowing maintained sufficient spacing between units.

We excluded designated areas for placing fire-exposed seeds in terracotta dishes (see 2.6) and for soil analysis from sowing. Seedling establishment was monitored throughout the growing season. Both fire and control plots were covered with bird nets to prevent disturbance by larger animals.

### 2.3 The experimental fire

Two weeks before the experimental fire, the fire plot was covered with a transparent plastic foil mounted 70 cm above the plot surface to withhold precipitation and simulate a dry period. For safety reasons, the fire experiment was planned on the first sunny day following a rainy period. On the day of burning, the rainout shelter was removed, and 10 L of additional dry litter (collected beforehand from the neighboring forests and dried at 80 °C for 48 h) was evenly distributed on the fire plot. This ensured homogeneous and fast ignition. The surface was then sprayed with ethanol and ignited at six points in the fire plot (11:13 am CEST, UTC + 02:00). The fire was allowed to burn for ca. 6 min, simulating a low- to moderate-intensity surface fire, before being extinguished with tap water. The fire resulted in a homogeneously burned area, with a black ash layer (burned litter and charred branch pieces) on top, an intermediate layer of partially burned litter, and an underlying unburned soil layer.

### 2.4 Microclimate during and after the fire

During the fire, air and soil temperatures were measured at 1-sec intervals using high-temperature type-K thermocouples. Air temperature sensors were installed at 5, 30, and 55 cm above the soil surface. Thermocouples of the same type were installed at depths of 2, 5, and 10 cm below the soil surface, as well as 1 cm below the soil surface within the dishes (see 2.6). All thermocouples were connected to temperature loggers (PCE-T390, PCE Instruments, Germany). Heat flux was measured at 1-sec intervals using a water-cooled heat flux sensor (SBG01, Hukseflux Thermal Sensors BV, the Netherlands) installed 55 cm above the soil surface and connected to a data logger (Campbell CR10X, Campbell Scientific, Ltd., UK).

After the fire, throughout the growing season, soil temperature (at 1 cm depth in the soil layer) in both plots was recorded at 10-min intervals using dataloggers (MicroLog SP and Minikin TT; EMS, Czech Republic). Precipitation data were recorded by a nearby weather station. On days when seedling establishment was assessed, surface temperatures in the fire plot were measured manually from morning to midday using four type-K thermocouples (three type-K thermocouples were used from DOY 219 onwards). All thermocouples were connected to a temperature logger (PCE-T390, PCE Instruments, Germany). The thermocouples were randomly positioned across the fire plot within the upper 1 cm of the ash layer.

### 2.5 Edaphic factors

Soil was sampled right before the fire and on each sowing date after the fire. Soil was sampled within the designated area in the fire plot (**Supplementary Figure 2**), resulting in one pre-fire sample collected from the soil on DOY 156 and five post-fire samples collected on each sowing date. After the fire, soil was sampled from three layers: burned litter and ashes at the surface (roughly 0–3 cm, henceforth referred to as ash), unburned litter (roughly 2–5 cm, henceforth referred to as litter), and the uppermost soil layer (roughly 5 cm, henceforth referred to as soil). For pre-fire sampling, only soil was sampled, as the other two layers were not present.

Soil pH and electrical conductivity (EC) were measured as three technical replicates from one sample by extracting 3 g of the respective material with 35 mL of distilled water and shaking for 22 h. Extracts were then filtered, and pH (826 pH mobile, Metrohm, Austria) as well as EC (LF 330/SET, WTW TetraCon, Xylem Analytics, Weilheim, Germany) were measured. On the same extraction solutions, analysis of 23 plant nutrient and toxic elements (Mg, K, Ca, Zn, S, Sr, P, Mn, B, Cu, Ni, Al, Fe, Na, Cd, Co, Cr, Li, Mo, Pb, Se, V and W) was performed to determine the concentration in plant available water in the soil, rather than overall concentration in the bulk material. Measurements were carried out using inductively coupled plasma optical emission spectrometry (ICP-OES) with a Spectro Genesis ICP-OES (AMETEK, Germany).

### 2.6 Seed germination

Germination tests were conducted to evaluate the maximum germination potential of the seeds and the effects of fire exposure on germination. Before the fire (on DOY 156), 50 seeds per species were placed in a terracotta dish filled with a 2-cm soil layer (ø13 cm for *L. decidua* and *P. cembra* mixed in the dish; ø11 cm for *P. abies*; 12 replicate dishes per setup). The dishes were randomly placed in the designated area within the fire plot at a depth of 3 cm (**Supplementary Figure 2**), with their upper edges positioned 1 cm below ground level. Finally, the dishes were covered with a 1-cm layer of soil. After the fire, dishes were removed, and the fire-exposed seeds were transferred to the laboratory for germination testing.

Germination tests were performed under controlled laboratory conditions two days after the experimental fire. Fire-exposed seeds of *P. cembra, P. abies*, and *L. decidua* were sieved, and any remaining debris was carefully removed before testing. Two hundred seeds per species were randomly selected from the fire-exposed plot, and 200 seeds per species from the non-fire-exposed plot served as controls. Two 85 mm Whatman filter papers were hydrated with 3 mL Milli-Q water in 90 mm polystyrene Petri dishes (VWR, USA). Subsequently, fire-exposed and control seeds were placed in the Petri dishes, which were then sealed with Parafilm. Seeds were arranged in five rows of ten seeds each, for a total of 50 seeds per Petri dish. Four replicates (n = 4) were conducted for each species and treatment. Optimal germination conditions were selected according to ISTA (2024), and seeds were germinated in a growth chamber (PGC-6HO, Percival Scientific, USA) at a daytime light intensity of 50 µmol quanta m^–2^ s^–1^. Germination was recorded daily for 22 days after sowing and subsequently at multi-day intervals from day 25 to day 32, until no further increase in total germination was observed. A seed was considered germinated when the radicle protruded through the surrounding seed covers by more than 2 mm. Total germination was calculated as the percentage of germinated seeds relative to the total number of seeds tested.

### 2.7 Seedling establishment

From DOY 156, the abundance of established seedlings was monitored every 10 days (the last two intervals were 12 and 11 days, respectively) in both plots in the field. The 10-day monitoring cycle finished on DOY 219, by which time the number of seedlings had stabilized. Two further assessments were conducted on DOY 235 and DOY 266. Intact seedlings with clearly visible cotyledons were counted as established seedlings. In addition, the number of seedlings displaying visible damage (e.g., wilting, broken stems, and missing needles or cotyledons) was recorded.

### 2.8 Seedling biomass and size

At the end of the growing season (DOY 266), all seedlings had fully developed and formed cotyledons. All seedlings were carefully uprooted, sealed in plastic bags, and transported to the laboratory for immediate biomass analysis. As Alpine conifer seedlings at these early growth stages exhibit little further height growth (Moser et al., 2010), we expected harvesting at the end of the experiment to enable a reliable comparison of their biomass and thus growth. For each sowing date, the three largest seedlings per species with no visible damage were selected from each of the three sowing units (i.e., n = 9 seedlings per species per sowing date). Their length, needle area, and dry weight were determined. In cases where fewer than nine seedlings had established from a given sowing date, all available seedlings were analyzed. Lengths of stems (including closed buds) and roots were measured with a caliper (BAHAG AG, Mannheim, Germany). Seedlings were then cut into roots, stems, and needles for the following measurements. The needle area was digitized with a scanner (bizhub C458, Konica Minolta, Austria) and analyzed using ImageJ image analysis software (https://imagej.net/ij/). Subsequently, all organs were oven-dried at 80 °C for 24 h. The dry weight of the needles, roots, and stem (including closed buds) was determined using an analytical balance (Sartorius BP61S, 0.0001 g precision; Sartorius AG, Germany).

### 2.9 Statistical analyses

Average daily soil temperatures were calculated for the entire measurement period. Peak temperatures (mean ± SE) at the soil surface were derived from the maximum values recorded by thermocouples installed in the ash layer. Differences in germination between control and fire-exposed seeds were tested for significance through ANOVA at each time point. Differences in EC and pH among the three post-fire layers (ash, litter, and soil), as well as post-fire versus pre-fire soil values, were tested using ANOVA. To model soil temperature parameters (surface temperatures, diurnal temperature fluctuations, as well as minimum and maximum soil temperatures) with DOYs, a linear regression was conducted for each parameter with the lm function in R (R version 4.4.2, R Core Team, 2024). Regression results were used to observe significant changes in changes (dependent variable) over time (independent variable). For seedling biomass, length, and needle area, normality was assessed using the Shapiro-Wilk test. Comparisons between seedlings from fire-exposed seeds or post-fire sowings and their respective control treatments were performed using unpaired *t*-tests when normality assumptions were met and Mann–Whitney tests otherwise. To test whether establishment in control and fire plots was significantly different from zero and whether establishment in the control plot was significantly higher than in the fire plot, general additive models (GAM) were used. Individual models for each of the three tree species and five sowing dates were calculated, which led to a total of 15 models. Establishment success was determined as the dependent variable, while DOY and fire/control plot served as continuous and categorical predictor variables, respectively. GAMs were conducted in R (R version 4.4.2, R Core Team, 2024) with the mgcv package (Wood, 2025) and the gam function, using thin plate splines with seven basis functions up to sowing on DOY 176 and five for DOY 186 and DOY 196, due to fewer available data points. SPSS (IBM SPSS 204 Statistics 21, IBM) was used to conduct ANOVA tests on laboratory germination data, while all other tests were conducted using R (R version 4.4.2, R Core Team, 2024). Significance was accepted at *p* ≤ 0.05.

## 3 Results

### 3.1 The experimental fire

**Figure 1:**
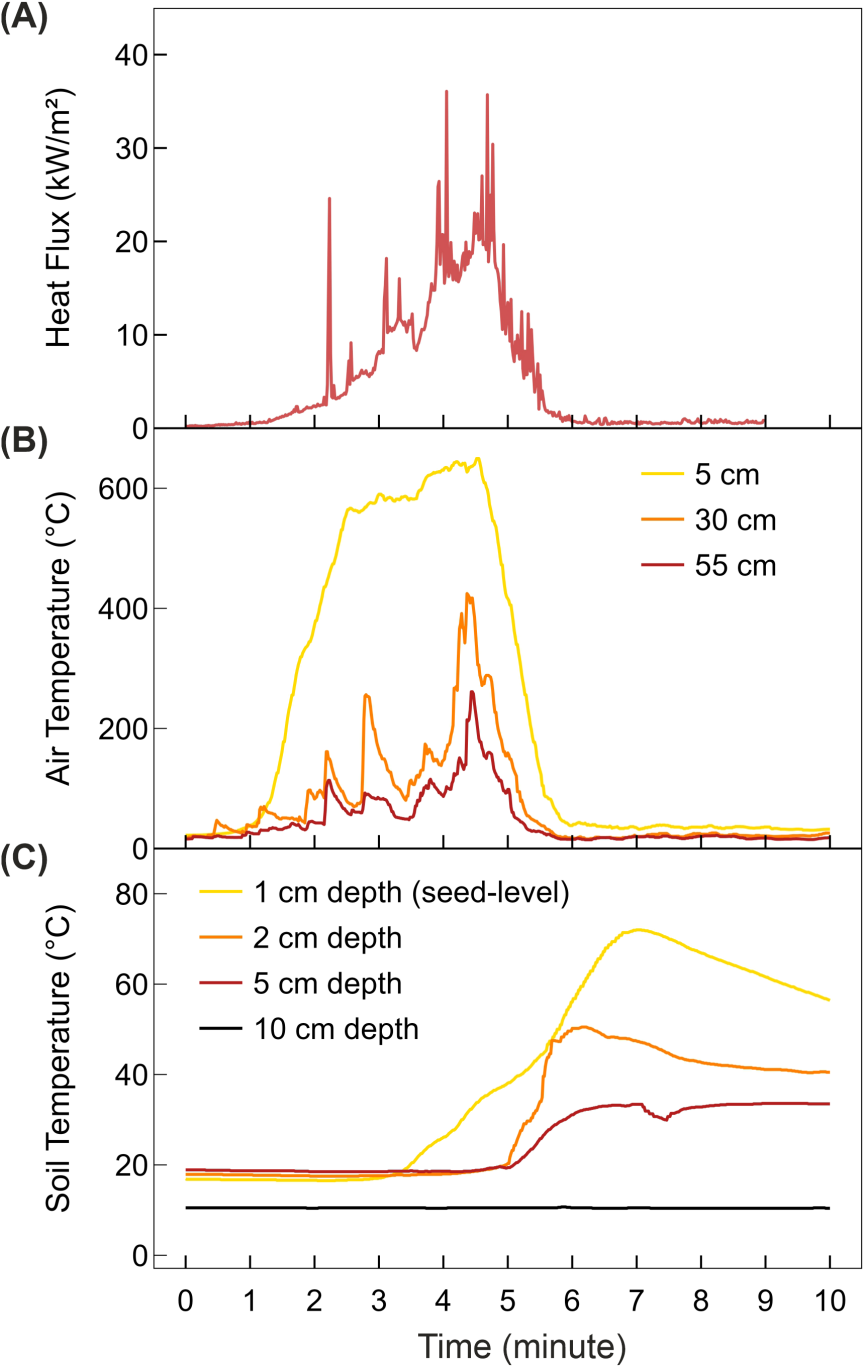
Conditions during the experimental fire. The fire was ignited at time point 0. **(A)** Heat flux **(B)** air temperature at different heights above the soil surface, with heights indicated by different colors; **(C)** soil temperature at different depths below the soil surface, with depths indicated by different colors.

The experimental fire lasted approximately 6 min. Approximately 4 min after ignition, a total heat dose of 1217.0 MJ/m² was released, with a peak flux of 36.1 kW/m² (**Figure 1A**). The highest air temperature was recorded 5 cm above the soil surface, reaching a maximum of 649.5 °C (**Figure 1B**). At this height, air temperature exceeded 500 °C for 2.5 min and 600 °C for 1 min. The highest soil temperature during the fire occurred at the seed level in the dishes (1-cm depth), reaching 72.0 °C. At 2-cm depth, soil temperature increased to 50.5 °C, whilst at 5-cm depth, temperature did not exceed 40 °C. At 10-cm depth, soil temperature was not affected (**Figure 1C**).

### 3.2 Post-fire edaphic conditions

**Figure 2:**
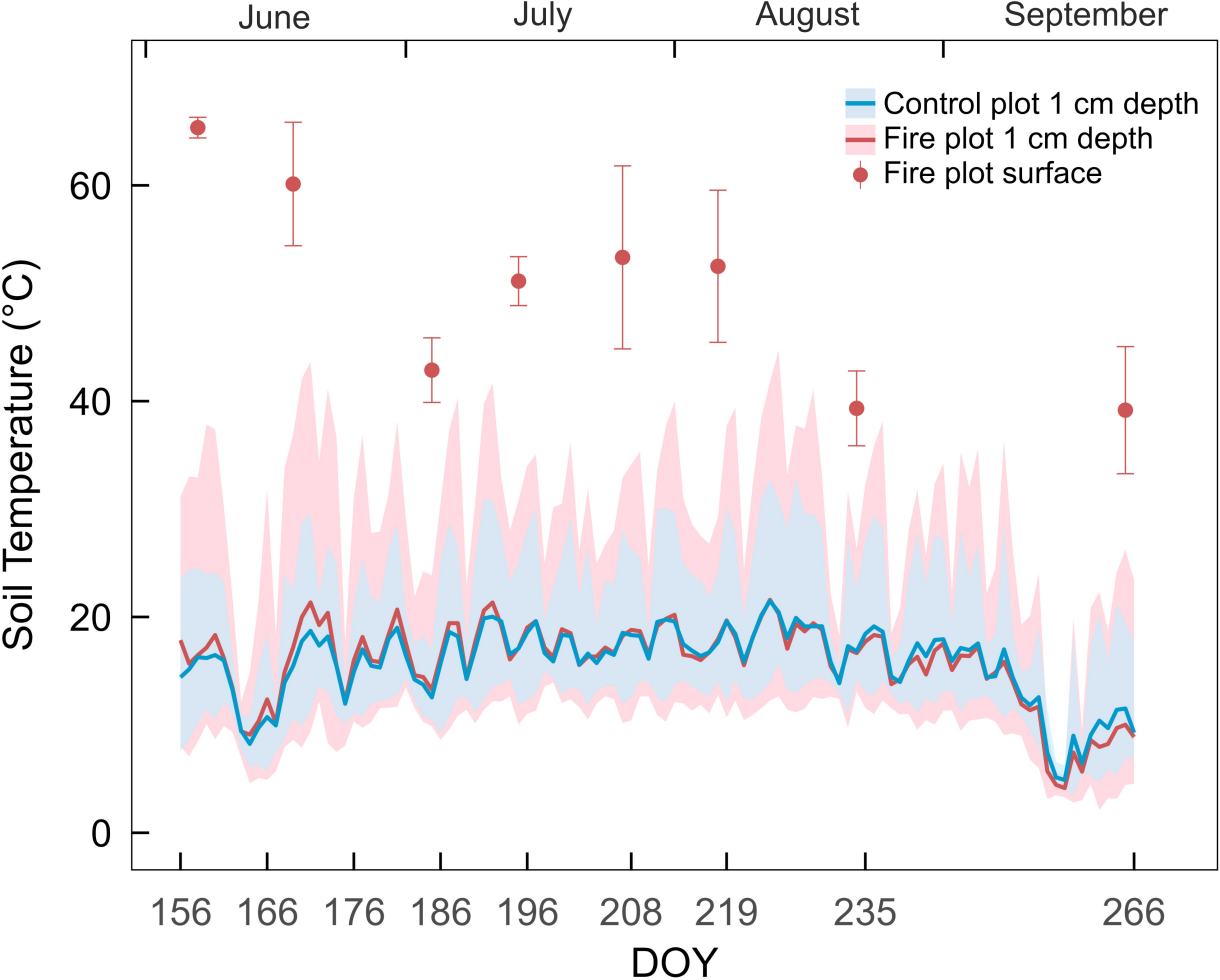
Soil temperature at a depth of 1 cm below the soil surface throughout the growing season in the fire plot (red) and control plot (blue). Solid lines indicate average daily temperatures, and shaded areas represent the daily temperature range (minimum to maximum). Red dots show peak temperatures at the soil surface of the fire plot, measured manually with thermocouples (on sunny days around midday, means ± SE).

During the first 20 days after the fire, mean soil temperature at 1-cm depth was up to 3.4 °C higher in the fire plot than in the control plot (**Figure 2**). Higher daytime and lower nighttime temperatures were recorded in the fire plot, resulting in larger daily temperature fluctuations compared to the control. Differences in daily temperature amplitude between the fire and control plots declined over time (0.05 K change per day, *p* < 0.001; **Supplementary Figure 3**). We found that the main reduction occurred in soil maximum temperature differences between the fire and control plots (0.05 K change per day, *p* < 0.001). Additionally, differences in soil minimum temperatures between the plots remained relatively stable (0.005 K change per day, *p* = 0.01). Maximum daytime temperatures in the fire plot exceeded 40 °C on eight of our measuring dates. The highest soil temperature occurred in August, reaching 44.7 °C in the fire and 32.8 °C in the control plot. The highest surface temperature in the fire plot was 65.4 °C, recorded in June. Surface temperatures in the fire plot declined significantly over time (0.2 K change per day, *p* = 0.017).

**Figure 3:**
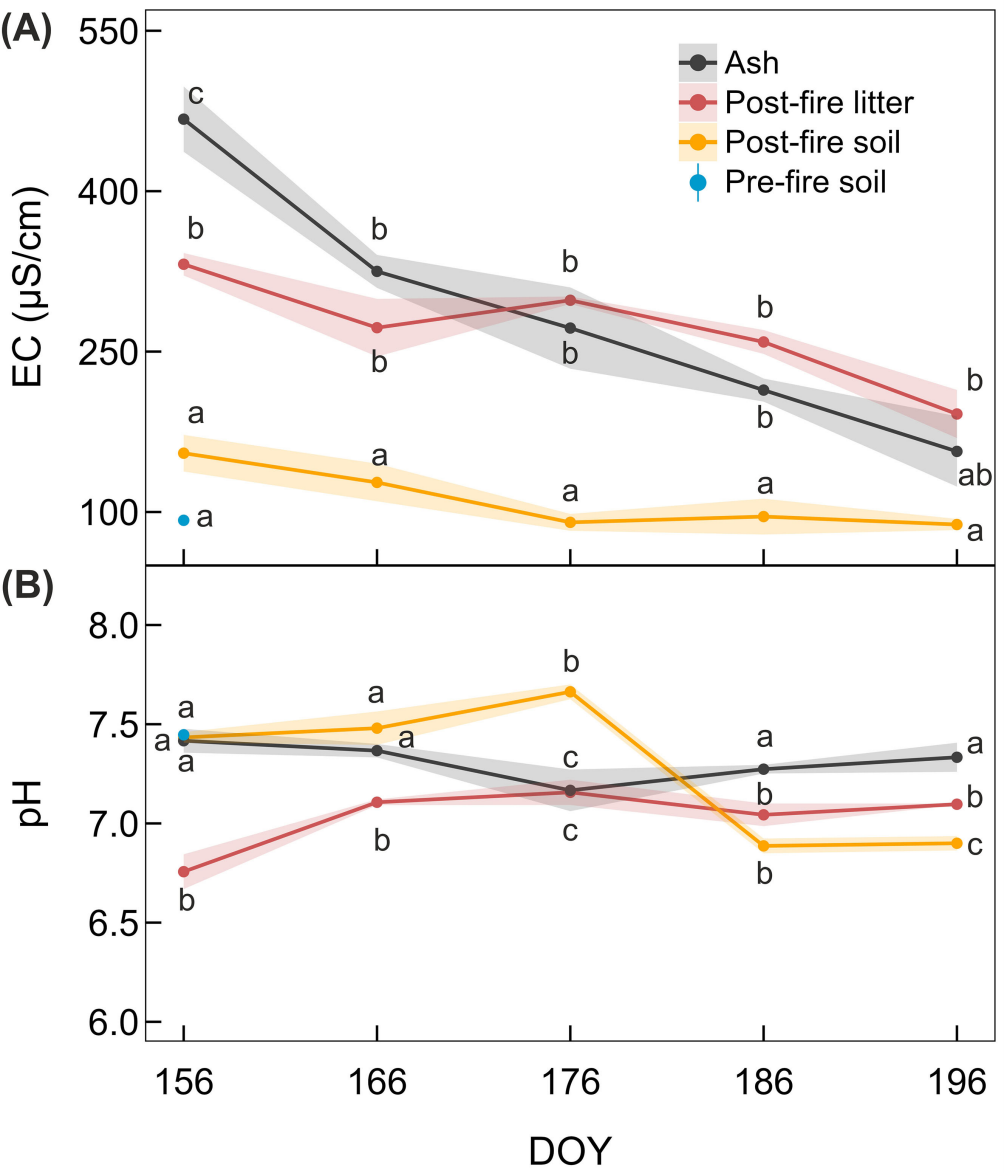
Effects of the experimental fire on edaphic factors. **(A)** Soil electrical conductivity (EC) and **(B)** pH in the pre- and post-fire soil components, with components indicated by different colors. Pre-fire soil samples (blue) from the fire plot were analyzed on day of the year (DOY) 156. Letters indicate statistical significance among post-fire layers (ash, litter, and soil) at each sampling day and pre-fire soil layer (on DOY 156), respectively. Data are means ± SE (shaded areas); triplicate measurements.

The average precipitation per sampling interval was 45 mm (sampling occurred from DOY 156 to 196, in 10-day intervals), with a slight increase during the summer period (**Supplementary Figure 4**). Repeated rain transported fine ash particles into the underlying litter layer and the soil beneath it during the following weeks after the fire. Therefore, the fire plot surface progressively changed to brownish colors over this time. EC in both the ash and litter layers was the highest directly after the fire and declined over the subsequent weeks (**Figure 3A**). By the final sampling date, EC in the ash layer had reached pre-fire soil values. Soil EC was slightly elevated after the fire; however, this was not statistically significant. In contrast, soil pH was not affected directly after the fire (**Figure 3B**) but decreased after DOY 176 from pH 7.7 to pH 6.9. The pH of the litter layer in the fire plot was initially lower than that of the soil (both pre- and post-fire) and ash layers; however, it gradually increased over time.

The ICP measurements indicated that nine elements exhibited trends similar to EC (**Supplementary Figure 5**), with initial increases and subsequent declines towards pre-fire levels, namely Mg, K, Ca, Zn, S, Sr, P, Mn, and B. Five elements showed no clear response pattern compared to pre-fire values and over time, namely Cu, Ni, Al, Fe, and Na. Nine of the tested elements (Cd, Co, Cr, Li, Mo, Pb, Se, V, and W) had concentrations below the detection limit of the instrument.

### 3.3 Germination under controlled laboratory conditions

**Figure 4:**
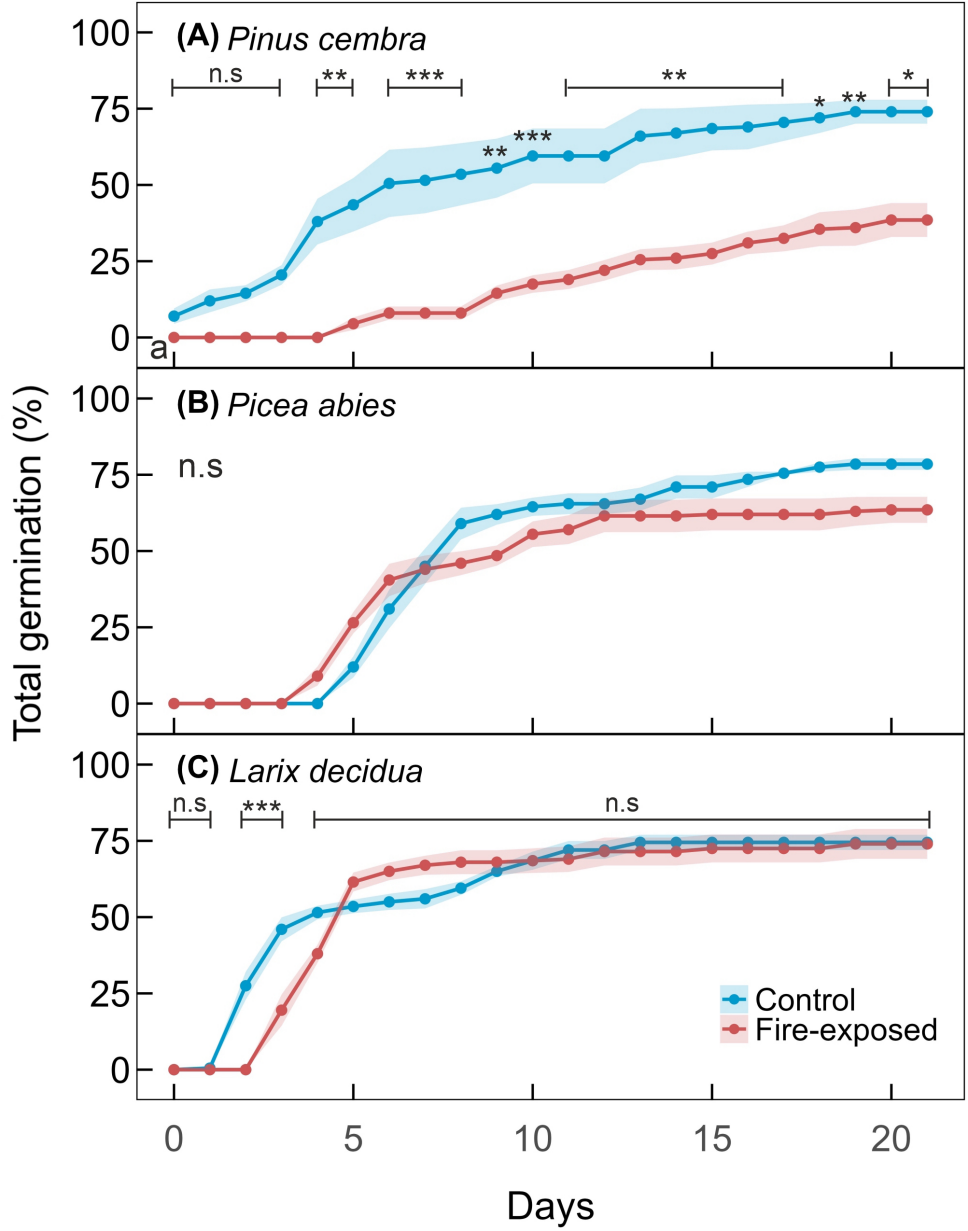
Germination of seeds under controlled laboratory conditions. The blue lines represent control seeds, and the red lines show fire-exposed seeds that were exposed above 60˚C for approximately 3 minutes. Data are means ± SE (shaded areas) of n = 4 replicates of 50 seeds each. Asterisks (*) denote statistical significance (*: *p* ≤ 0.05, **p ≤ 0.01, ***p ≤ 0.001). Data under the same level of significance are grouped.

Control seeds showed high total germination of 74% for *P. cembra*, 78.5% for *P. abies*, and 74.5% for *L. decidua*. Germination of fire-exposed *P. cembra* seeds was reduced by 35.5% compared to the control (**Figure 4A**), with a significant difference appearing from day 4 onward. In contrast, germination of fire-exposed seeds of *P. abies* and *L. decidua* was only slightly affected, with reductions of 15% and 0.5%, respectively (*p* > 0.05, **Figures 4B, C**). Initially, control seeds of *P. cembra* germinated faster than *P. abies*, while fire-exposed seeds showed overall slower germination and greater variation in the time until germination. In contrast, most *P. abies* and *L. decidua* seeds from both treatments germinated within one week after sowing.

### 3.4 Seedling establishment in the fire and control plot

**Figure 5:**
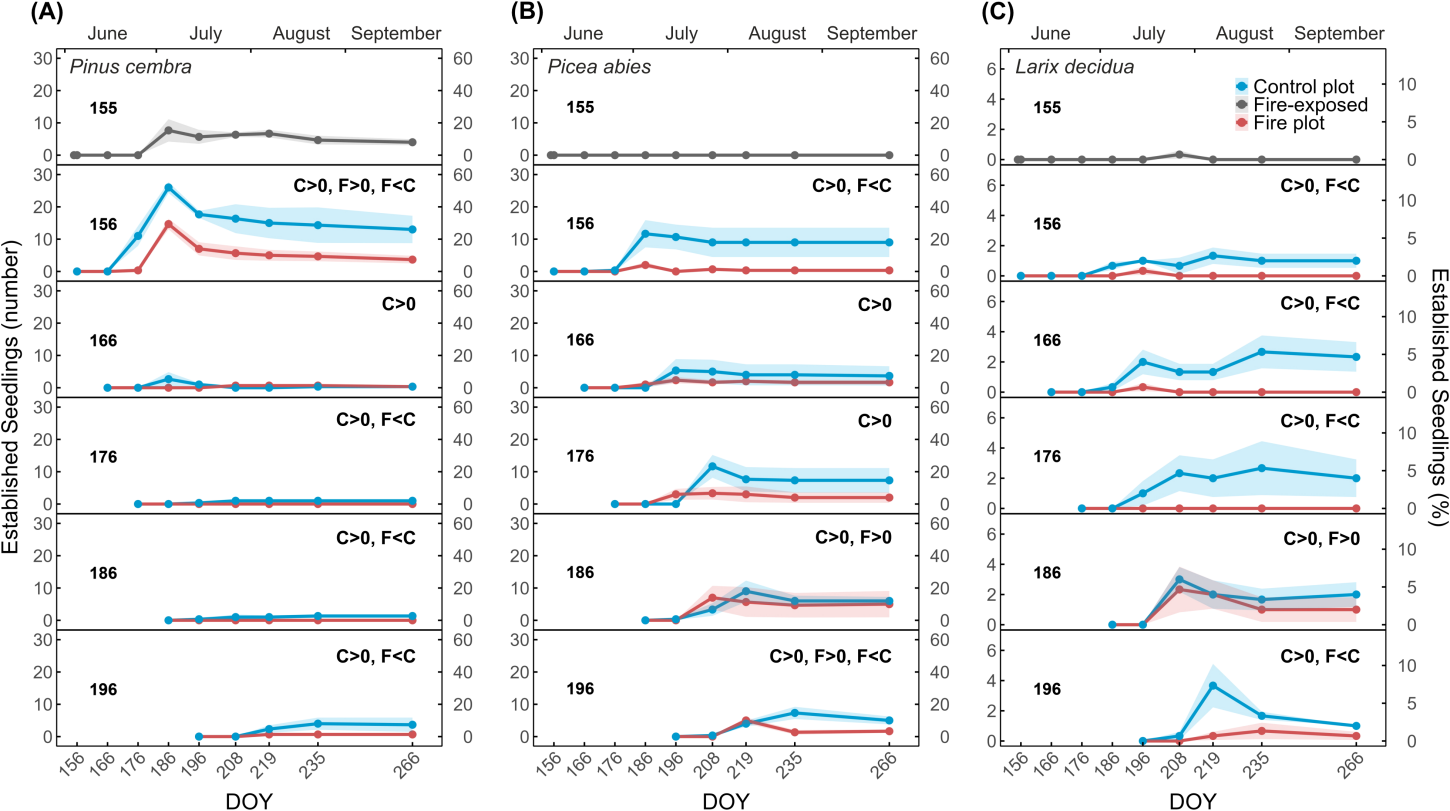
Seedling establishment in the fire and control plot for *P. cembra* **(A)**, *P. abies* **(B)**, and *L decidua* **(C)**. Bold numbers in panels indicate the days of the year (DOYs) on which seeds were sown. Curves show established seedlings (absolute and relative number in %) from fire-exposed seeds (top panels, grey) and from seeds sown on the control plot (blue) and the fire plot (red). Significance codes in bold (“F” denotes fire plot and “C” denotes control plot) in the top right corner of each panel indicate whether establishment was significantly higher than 0 for the fire (F>0) and control plot (C>0), when establishment in the fire plot was not significantly higher than 0, no code is shown. F<C denotes significantly lower establishment in the fire plot compared with the control, based on the GAM output; if no difference between plots was detected, no code is shown. Data are means ± SE (shaded areas).

Seeds exposed to fire exhibited a pronounced reduction in seedling establishment. No seedlings established from fire-exposed *P. abies* or *L. decidua* seeds. *P. cembra* showed an establishment of 15.3% by DOY 186, and 8% survived to the end of the season. This corresponded to 52.0% and 26.0%, respectively, of the establishments in the control plot (**Figure 5**, upper panels).

For all post-fire sowing dates (beginning on DOY 156), seedling establishment was lower in the fire plot than in the control plot for all species. GAM models detected significantly lower establishment in the fire plot for four sowing dates for *P. cembra*, two of *P. abies*, and four of *L. decidua* (**Supplementary Table 2**). Among tested species, *P. cembra* reached the highest establishment from seeds sown immediately after the fire (29.3% in the fire plot and 52.0% in the control plot; **Figure 5A**). However, final seedling establishment of *P. cembra* declined over time, and later sowing resulted in strongly reduced establishment in both plots. According to the GAM output, seedling establishment from seeds sown after DOY 166 remained low but significantly different from zero in the control plot (*p* < 0.001), whereas establishment in the fire plot was not significantly different from zero (**Supplementary Table 2**). A small increase was observed for seeds sown in late summer (DOY 196), as establishment percentage increased again slightly in the control plot. *P. abies* showed a lower establishment (maximum of 23.3% on the control plot from seeds sown at DOY 156 and 176), but more seedlings were also able to establish from later sowing dates (**Figure 5B**). The highest final establishment percentage in the fire plot (10.0%) was recorded when seeds were sown one month after the fire. Early in the season, establishment for *P. abies* in the fire plot was significantly lower than in the control, with differences decreasing over time. For seedlings sown on DOY 156, establishment in the fire plot was significantly lower than in the control plot (*p* < 0.001, **Supplementary Table 2**). Although establishment on sowing dates DOY 166 and 176 did not differ significantly between plots, establishment under post-fire conditions tended to be reduced and was not significantly different from 0. On sowing date DOY 186, establishment in the fire plot was statistically higher than 0 (*p* < 0.01) and did not differ from the control plot. *L. decidua* showed the lowest establishment among species (**Figure 5C**). Its highest establishment occurred when seeds were sown on DOY 186 in the fire plot (4.7%) and on DOY 196 in the control plot (7.3%). Differences between plots were again more pronounced at early sowing dates (*p* < 0.01), and only seeds sown on DOY 186 showed establishment in the fire plot that was significantly different from zero (*p* < 0.05) and statistically equal to the control (**Supplementary Table 2**).

### 3.5 Seedling size and biomass

At harvest (DOY 266), all seedlings were fully developed and with formed cotyledons. Seedlings established from fire-exposed seeds were observed only in *P. cembra*. Compared with control seedlings (sown on DOY 156), seedlings from fire-exposed seeds had slightly lower total biomass (by 0.006 g) and needle area (by 0.53 cm^2^), whereas stem length was significantly higher, by 2.17 cm (*p* < 0.001).

**Figure 6:**
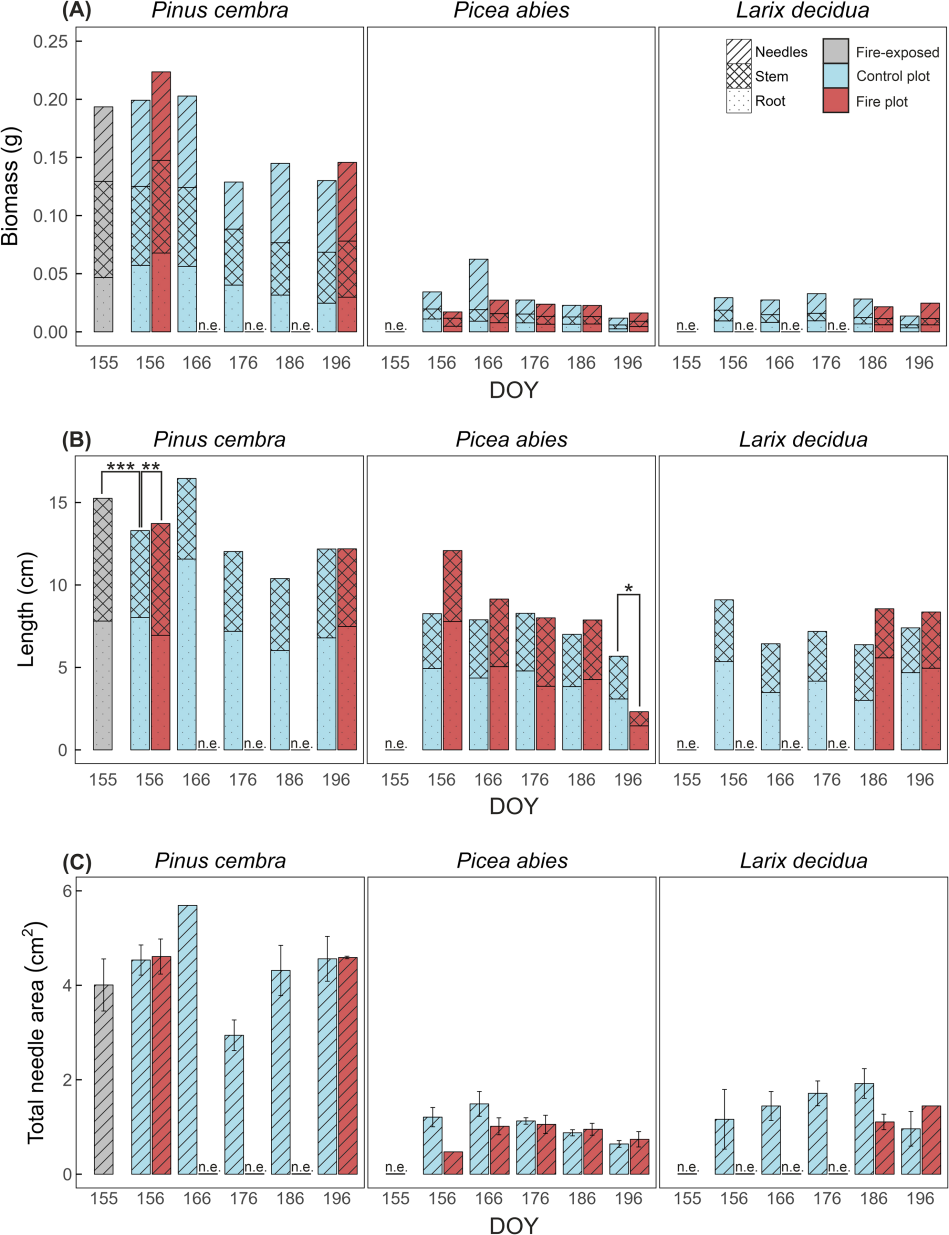
Effects of the experimental fire on biomass, stem and root length, and needle area of established seedlings. Seedlings derived from seeds exposed to the fire (grey) as well as from seeds sown on the fire plot (red) and control plot (blue) at different days of the year (DOY). All seedlings were harvested on DOY 266. ‘n.e.’ indicates no seedling established from the corresponding sowing date. **(A)** Biomass of needles, stem, and roots (g), **(B)** length of stem and roots (cm), **(C)** total needle area (cm²). Means **(A, B)** or means ± SE **(C)**. n = 9 replicate measurements; all available seedlings were analyzed when fewer than nine seedlings had established. Asterisks (*) indicate statistical significance (*p ≤ 0.05, **p ≤ 0.01, ***p ≤ 0.001).

Control *P. cembra* seedlings had overall lower biomass and root length when sown later in the growing season (**Figure 6**), while stem length and needle area showed no consistent trend. Among the studied species, *P. cembra* seedlings had the highest biomass (up to 0.224 g per plant). When sown on DOY 156 in the fire plot, established *P. cembra* seedlings showed slightly higher total biomass (0.02 g difference) and needle area (0.07 cm^2^ difference) compared with the control, with stem length being significantly longer (by 1.51 cm, *p* < 0.01).

In *P. abies*, control seedlings exhibited higher biomass and needle area at earlier sowing dates, followed by a decline after DOY 166 (up to 0.062 g and 1.49 cm^2^ per plant). In the fire plot, this decline was delayed and less pronounced. Seedling stem and root length also declined with later sowing in both plots, although the decline occurred earlier and was more pronounced in the fire plot. Seedlings from seeds sown on DOY 196 had the shortest stem and root length in the fire plot, with stem length significantly reduced compared with the control plot (by 1.73 cm; *p* < 0.05).

In *L. decidua*, control seedlings showed higher biomass and needle area at later sowing dates (≥ DOY 176; up to 0.033 g and 1.92 cm^2^ per plant), followed by a decline for seedlings sown on DOY 196. The longest total length (9.10 cm) was found in the control seedlings sown on DOY 156. In the fire plot, seedlings only established from later sowing dates (DOY 186 and 196). Compared with the control, seedlings sown on DOY 186 in the fire plot exhibited slightly lower biomass and smaller needle area (by 0.007 g and 0.82 cm^2^, respectively); however, seedlings sown on DOY 196 showed higher values (0.011 g and 0.49 cm² higher, respectively). Additionally, seedlings sown on DOY 186 and 196 in the fire plot developed higher total length than the control (2.18 and 0.96 cm difference, respectively).

## 4 Discussion

Our experiment successfully simulated a low- to moderate-intensity surface fire typical of Alpine conifer forests (Bradstock and Auld, 1995; Caon et al., 2014; Neary et al., 1999; Scott et al., 2014). As expected, overall seedling establishment markedly decreased in the burned plot due to unfavorable conditions regarding surface temperature, water supply, and edaphic conditions. After fire exposure, only *P. cembra* seeds developed into seedlings. *P. abies* and *L. decidua* failed to establish seedlings, although germination was not affected by fire exposure. When sown directly after the fire, *P. cembra* exhibited the best seedling establishment. Over the entire growing season, *P. abies* achieved the most successful establishment overall, while *L. decidua* showed the least establishment.

### 4.1 Environmental conditions during and after the experimental fire

The fire simulated in this experiment and the temperature conditions experienced by seeds closely resembled those of natural surface fires. The maximum air temperature induced by the experimental fire reached 649.5 °C (**Figure 1B**), which was in line with the 150–700 °C range recorded for surface fires in coniferous forests (DeBano et al., 1976; Habrouk et al., 1999; Rundel, 1983). In the present study, the soil temperature peaked at 72 °C, and the upper soil layer (where seeds were positioned; see 4.2) exceeded 60 °C for approximately 3 minutes (**Figure 1C**). Published soil temperature profiles for low- to moderate-intensity wildfires also demonstrated peak temperatures of 50 to 100 °C in the top 5 cm of soil (Bradstock and Auld, 1995; Caon et al., 2014).

Following fire, dark ash layers formed by combustion of organic matter strongly absorb solar radiation, causing substantial daytime heating of the upper soil (Jin et al., 2012; Randerson et al., 2006; Scott et al., 2014; Zhao et al., 2024). Accordingly, in our study, long-term increases in soil surface temperatures were observed in the fire plot (**Figure 2**), with peak temperatures exceeding 65 °C and pronounced daily temperature amplitudes (Liu et al., 2018; Zhao et al., 2021). High surface temperatures in the fire plot likely increased evaporation, resulting in drier litter and upper soil layers (Williams et al., 2013). Wildfires can also increase soil hydrophobicity (Weninger et al., 2019), creating a water-repellent upper layer (Agbeshie et al., 2022; Bodí et al., 2011; Scott et al., 2014). Consequently, post-fire conditions were characterized not only by harsh temperature regimes but also by dry conditions, particularly in the upper soil layers. These effects diminished over time when the dark ash layers were washed out (**Supplementary Figure 4**) and led, for example, to decreasing daily temperature amplitudes over summer (**Figure 2** and S3). Immediately after the fire, EC was five times higher than pre-fire levels (**Figure 3A**), consistent with reports of up to a seven-fold increase in other studies (del Pino et al., 2008; Granged et al., 2011). This is likely caused by the accumulation of non-volatile cations in the ash (Alcañiz et al., 2016; Chen and Shrestha, 2012; Elliott et al., 2013; Rahimi et al., 2020). Over the summer, EC decreased to pre-fire levels because water-soluble cations and small organic molecules were leached during precipitation (Alcañiz et al., 2016; **Supplementary Figure 4**). Accordingly, EC decreased faster in the ash than in the litter. Ion concentrations mirrored EC patterns, with Mg, K, and Ca dominating the post-fire cation pool and likely driving the salinity changes (**Supplementary Figure 5**). Although none of the ions that increased in concentration are known to have toxic effects on plants, increased salinity can reduce soil water availability by lowering soil osmotic potential.

Interestingly, no changes in soil pH were induced by the fire (**Figure 3B**). This was likely due to the moderate soil temperatures during combustion compared with those observed in more severe wildfires (Agbeshie et al., 2022; Certini, 2005; Knicker, 2007; Rahimi et al., 2020). Similar stability in soil pH following burning has also been reported in previous studies (Alcañiz et al., 2016; Badía et al., 2014). The slightly acidic pH of the litter layer found in this study is typical for wood particles (Sandermann and Rothkamm, 1959). The moderate increase in litter pH over time was again caused by precipitation-driven leaching, which may have also caused the decrease in soil pH at the end of June (**Figure 3**).

### 4.2 Germination of fire-exposed seeds

*P. abies* and *L. decidua* seeds were relatively resistant to the experimental fire, while *P. cembra* showed a 35.5% reduction in germination (**Figure 4**). The large, oil-rich *P. cembra* seeds (~230 mg per seed; Grodziński and Sawicka-Kapusta, 1970) contain substantial nutrient reserves but are not very heat resistant (Muscolo et al., 2002; Sidari et al., 2008). The high oil content may favor rapid temperature increases in seed tissues, as oil possesses a relatively low heat capacity. In addition, the upper part of the large seeds was probably exposed to the fire when litter and upper soil layers were combusted, while the small seeds of *P. abies* and *L. decidua* (~6.7 mg and ~3.7 mg per seed, respectively; Grodziński and Sawicka-Kapusta, 1970) remained sufficiently buried in the soil. As small seeds were more easily covered by soil, *P. abies* and *L. decidua* may be better protected during surface fires than the larger seeds of *P. cembra*. However, these advantages may be counterbalanced by the subsequent seedling establishment under post-fire conditions (see 4.3).

### 4.3 Seedling establishment

Environmental conditions described above likely affected post-fire seedling establishment in the following ways. Firstly, seedlings were exposed to high temperatures and pronounced temperature fluctuations (**Figure 2** & S3). Heating is particularly critical for small seedlings in the near-surface boundary layer, where limited convective air exchange and full radiation (Baltzer et al., 2021; Meinzer et al., 2011) cause needle and stem temperatures to rise above ambient air temperature by 5 to 20 °C, as observed in *Pinus* and *Picea* spp. (Christersson and Sandstedt, 1978; Hadley and Smith, 1987; Harvey et al., 2016; Martin et al., 1999). Elevated needle temperatures can impair photosynthesis (Epron, 1997; Gette et al., 2020; Sage et al., 2008), carbon balance (Lindroth et al., 1998; Zha et al., 2001), and seedling growth (Zhao et al., 2013). Excessive heat is also reported to damage tissues (for conifers, see e.g., Baker, 1929; Helgerson, 1990; Kolb and Robberecht, 1996; Seidel, 1986). This may be especially relevant in the lower stem and upper root sections, which are in contact with the dark ash.

Secondly, developing seedlings were likely exposed to drought stress. High temperatures caused by dark ash layers likely intensified evaporation and thus reduced the surface soil moisture content relevant for seedlings. Simultaneously, increased salinity further limited the soil water available for plants. In parallel, root water uptake and transport capacity may be impaired due to heat damage, amplifying low water availability (Jagadish et al., 2021; McDowell et al., 2008). However, surviving seedlings in this study showed root biomass and length similar to controls (**Figure 6**).

Thirdly, the experimental fire changed ion concentrations and nutrient balance in the soil (**Figure 3** & S5). As none of the elevated ions identified by ICP measurements have toxic effects on plants (Angulo-Bejarano et al., 2021; Kučera et al., 2008), seedling establishment in the fire plot was more likely constrained by elevated salinity and reduced osmotic potential, as observed in previous studies (Alcañiz et al., 2016; Fernandez-Marcos, 2022; Steudle, 2000). As hydraulic functioning often determines whether seedlings survive and grow (Grossnickle and Ivetić, 2022), such osmotic stress may critically inhibit root growth and limit water uptake (Grossnickle and Ivetić, 2022; Lu and Fricke, 2023). In previous studies, EC values above 4000 µS/cm (4 dS/m) have been found to decrease survival rates of *Pinus sylvestris* and *Picea pungens* seedlings (Werkhoven et al., 1966) and growth of *Sequoia sempervirens* saplings (Nackley et al., 2015). Even with rather conservative 1:15 EC conversion of our 3:35 (w/v) aqueous extracts and saturated paste extracts (Alves et al., 2022), many ash and litter samples were above this threshold (266 µS/cm in our scale).

Seedling establishment from seeds exposed to the experimental fire was not possible in *P. abies* and *L. decidua* and was substantially reduced in *P. cembra* compared with the control plot (**Figure 5**). As germination tests revealed heat damage only in the latter, it is evident that seedling establishment was affected by post-fire conditions and not by the fire itself. *P. cembra* may benefit from its relatively larger seeds, which increases independence of seedlings from soil resource supply during the first days of germination (Dalling and Hubbell, 2002; Meinzer et al., 2011), even though these reserves may have been partly damaged by the fire. Compared with germination tests, which showed a reduction of 35.5% in fire-exposed seeds, seedling establishment was reduced by only 22.7% compared with controls (with overall lower establishment in controls than the germination potential), indicating pronounced limitation by abiotic and biotic factors during establishment. Surviving *P. cembra* seedlings had slightly lower biomass and needle area but significantly longer stems than controls (**Figure 6**), possibly required for growth through the ash layer.

Seedling establishment from non-fire-exposed seeds sown after the fire differed from that of fire-exposed seeds. For example, *P. cembra* seeds sown on the day of the fire already exhibited higher seedling establishment than seeds exposed to the fire. Post-fire sown seeds likely had a slightly higher water content (as the fire-exposed seeds were dried during the fire). Additionally, soil structure changed during the first days after the fire (e.g., collapse of ash and litter layers), and manipulation for seed placement in the subplots may have improved seed embedding in the soil. Thus, establishment of seedlings from post-fire sown seeds might not be directly comparable to that from fire-exposed seeds. *P. cembra* and *P. abies* seeds had a maximum establishment of 29.3% and 14%, respectively, while establishment in *L. decidua* reached only 4.7% (**Figure 5**). Again, the large seeds of *P. cembra* probably enabled seedling development relatively independent of soil nutrients and rapid formation of a larger root to reach soil water. However, high establishment percentages were only reached when *P. cembra* seeds were sown early in summer and at the end of the experiment. A similar trend was observed in the control plot (**Figure 5**). This likely reflects a generally reduced establishment under mid-summer conditions.

Contrastingly, *P. abies* and *L. decidua* exhibited increasing (although overall lower) establishment with the ongoing season. In the weeks following the fire, unfavorable conditions likely prevented seedlings from small seeds from developing sufficiently rapid shoot and root growth to access deeper soil water and develop stable, heat-tolerant tissues (Grossnickle, 2012; Johnstone and Chapin, 2006). Observed decreases in the number of established seedlings indicated that, despite successful initial growth, seedlings died off due to abiotic conditions. Additionally, as this experiment was conducted under field conditions, herbivores (e.g., slugs) may have further reduced establishment success. Seed size may be particularly relevant in the case of *L. decidua*, which has the smallest seeds and showed the lowest establishment. *L. decidua* seedlings are known to inherently depend on sufficient water availability (Mariani et al., 1990; Tiebel and Karge, 2025), rendering this species most susceptible to depleting its limited reserves under post-fire stress.

Interestingly, established seedlings of all studied species hardly differed in size or biomass between the fire and control plots. Therefore, once seedlings successfully established, their growth performance appeared largely predetermined, which is in line with the findings of Calvo et al. (2016). From an applied perspective, *P. cembra* could be the most promising species for sowing immediately after a forest fire, unless the fire occurs during warm summer months. Several weeks after fire, sowing of *P. abies*, potentially including *L. decidua* to increase tree species diversity, may represent an efficient alternative strategy.

## 5 Conclusion and outlook

Our study demonstrates that seeds exposed to fire, as well as temperature, hydraulic, and edaphic conditions after fire, substantially limit seedling establishment. Responses were species-specific regarding the extent and the dynamics of limitations: *P. cembra* achieved the highest establishment when seeds were sown directly after the fire, while *P. abies* showed the best overall establishment when sown four weeks later. Thus, our study provides the first insights into species-specific temporal dynamics of seedling establishment in Alpine conifers after fire, underscoring the importance of both species selection and timely sowing in post-fire forest management. Follow-up experiments should consider different situations regarding fire regimes (timing, fire intensity, and burned area), edaphic conditions, and post-fire climatic conditions to better predict the dynamics of seedling establishment and survival of Alpine tree species after fire disturbances. This will help determine the effective combination of technical and bioengineering measures for developing optimal post-fire reforestation strategies in Alpine forests.

## Data Availability

The original contributions presented in the study are included in the article/**Supplementary Material**. Further inquiries can be directed to the corresponding authors.
