# Supplementary material for: Insights into post-fire establishment of three Alpine conifer species after an experimental fire in Tyrol, Austria
Source: Front Plant Sci. 2026 Mar 17;17:1771923. doi: 10.3389/fpls.2026.1771923 (PMC13035797; doi:10.3389/fpls.2026.1771923)
Supplement: Supplementary file 1 [file Image1.pdf]

(A)

(B)

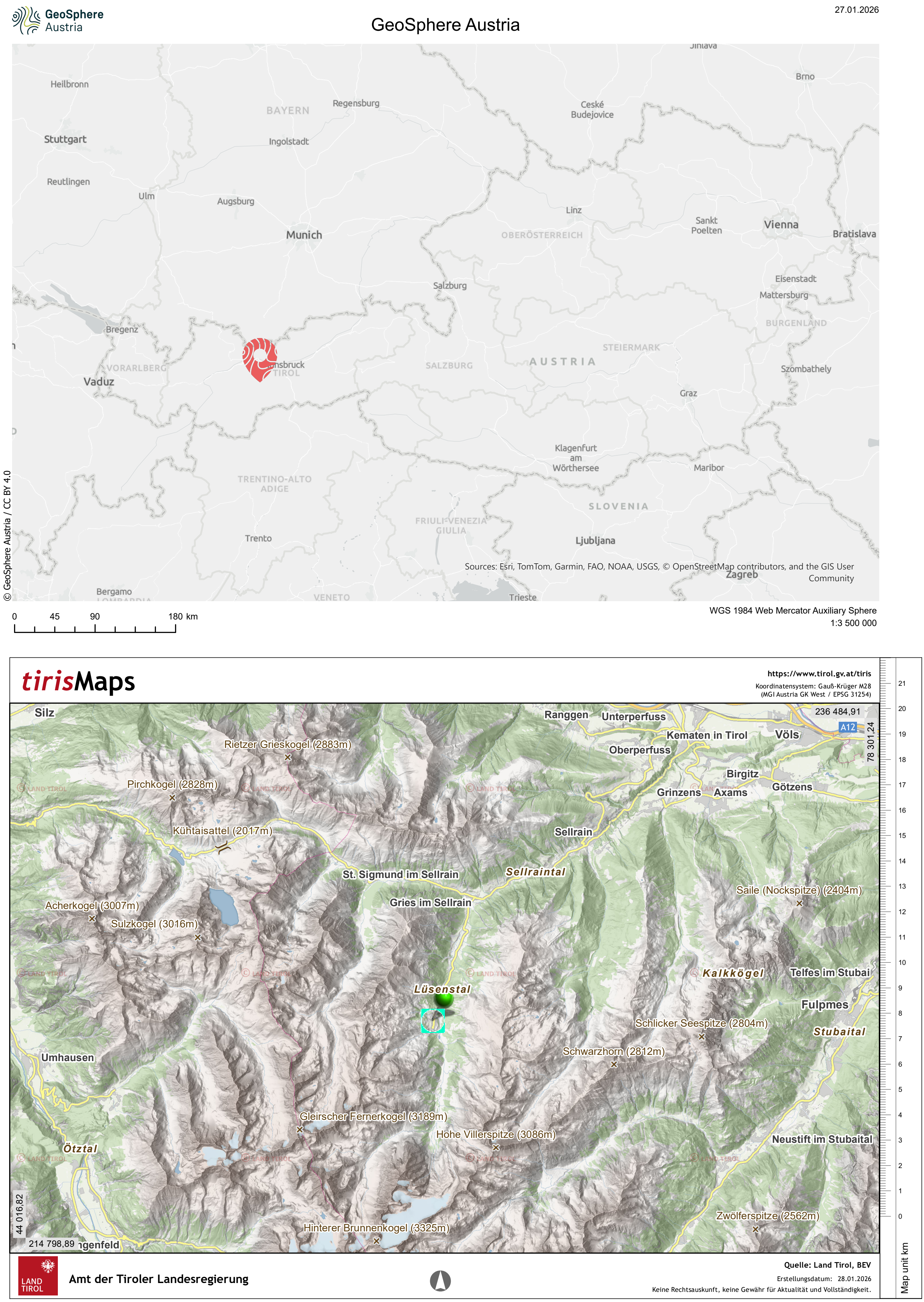

Figure S1 The geographical location of the experimental site Praxmar at both large **(A)** and small scales **(B)**. The study site is highlighted by placemarks. Data source: Geosphere and tirisMaps (accessed 27 January 2026).
