# Supplementary material for: Insights into post-fire establishment of three Alpine conifer species after an experimental fire in Tyrol, Austria
Source: Front Plant Sci. 2026 Mar 17;17:1771923. doi: 10.3389/fpls.2026.1771923 (PMC13035797; doi:10.3389/fpls.2026.1771923)
Supplement: Supplementary file 2 [file Image2.pdf]

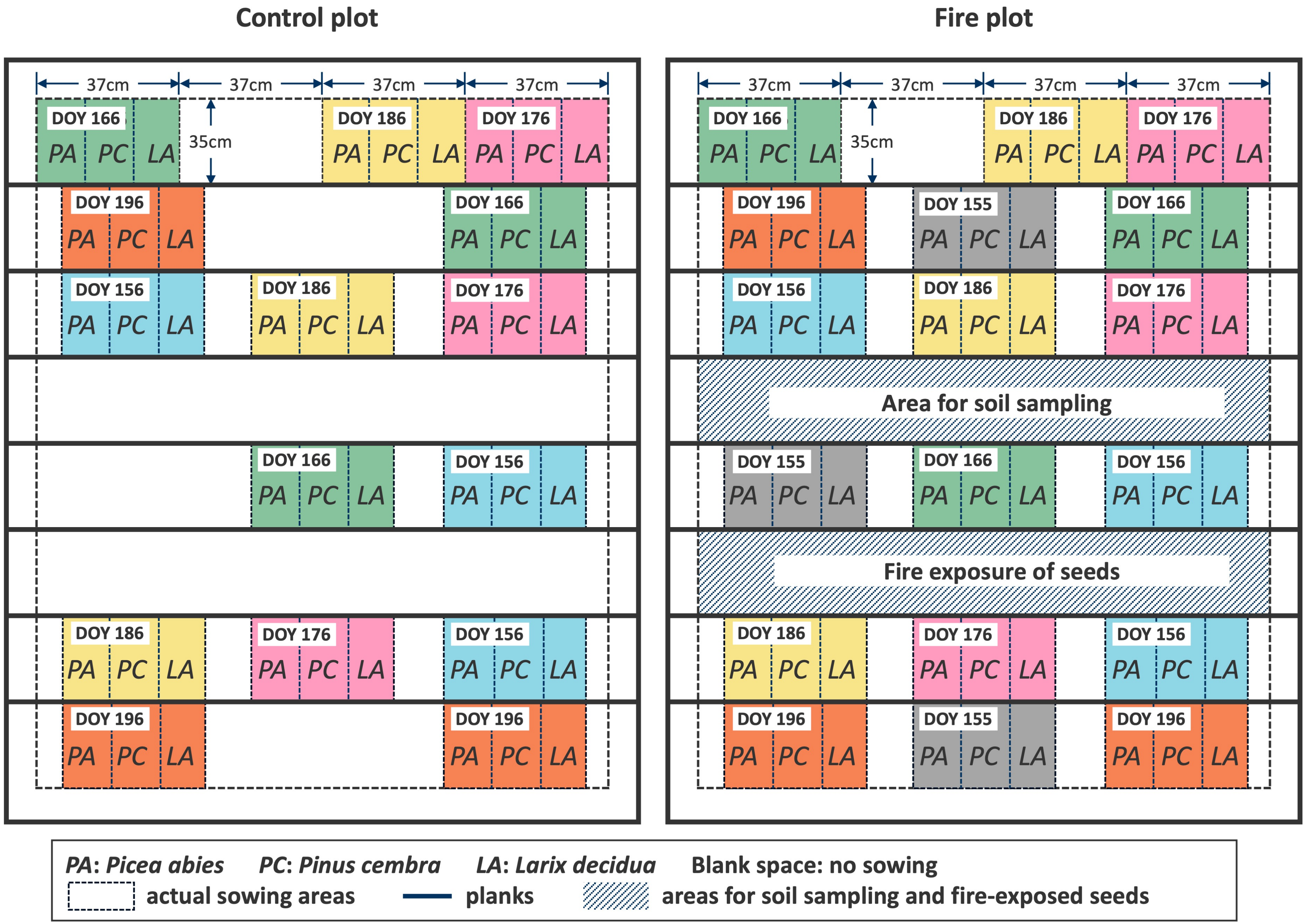

Figure S2 Design of the fire and control plots for sowing seeds from DOY (day of the year) 155 to DOY 196. Boxes with the same color represent identical sowing dates. The blank space remained empty from the seeds. Every sowing unit was separated into three columns of equal width, and each column contained one species. Seeds were sown in lines. Additional areas were used for soil sampling and for fire exposure of seeds.
