# Supplementary material for: Insights into post-fire establishment of three Alpine conifer species after an experimental fire in Tyrol, Austria
Source: Front Plant Sci. 2026 Mar 17;17:1771923. doi: 10.3389/fpls.2026.1771923 (PMC13035797; doi:10.3389/fpls.2026.1771923)
Supplement: Supplementary file 3 [file Image3.pdf]

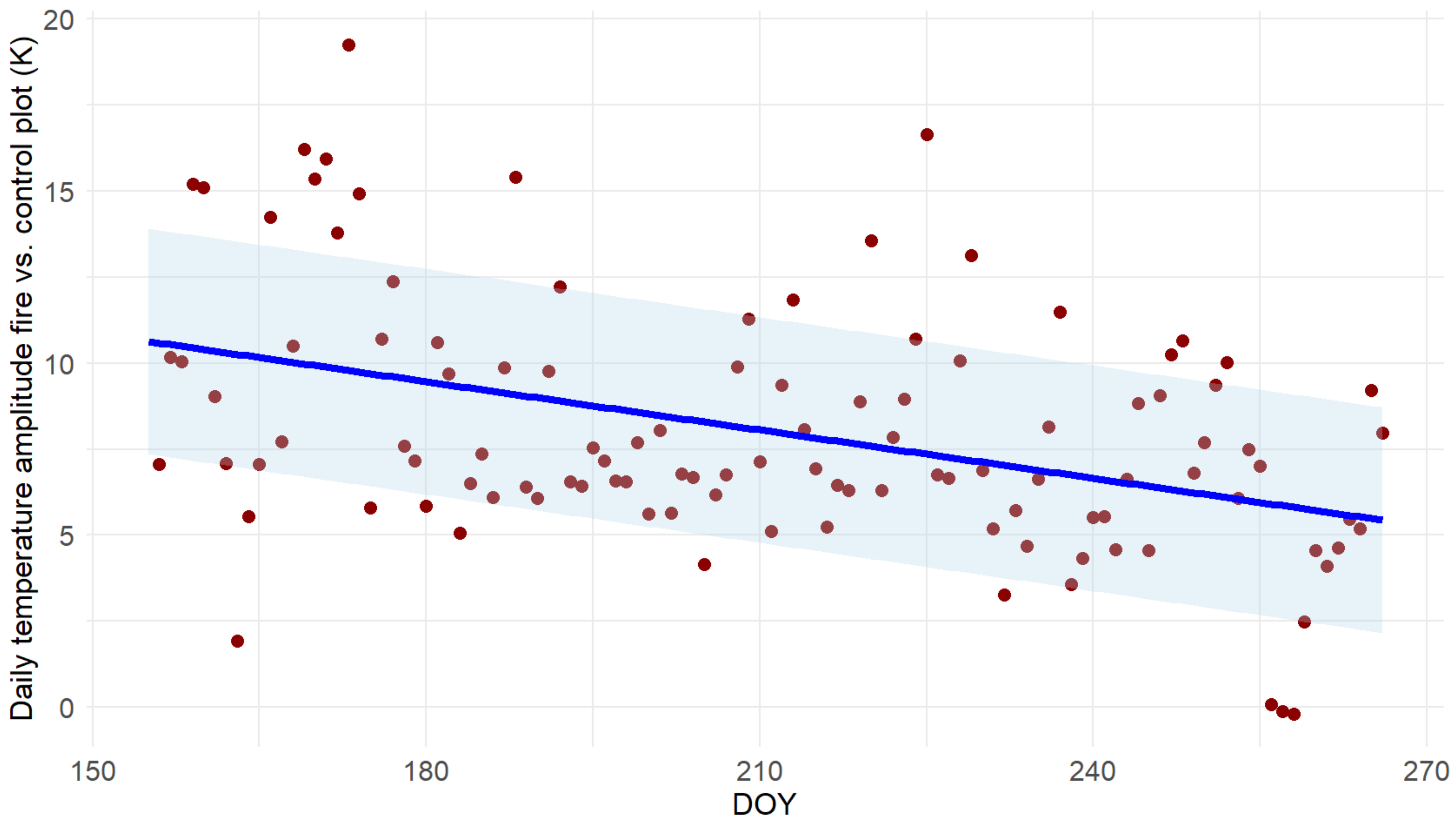

Figure S3 Difference between the diurnal temperature amplitudes of the control and fire plot over time. Points indicate the daily temperature amplitude between control and the fire plot. The blue line represents the linear regression ($y = 17.88 + 0.047 \times x$), data are means ± SD (shaded area).
