# Supplementary material for: Insights into post-fire establishment of three Alpine conifer species after an experimental fire in Tyrol, Austria
Source: Front Plant Sci. 2026 Mar 17;17:1771923. doi: 10.3389/fpls.2026.1771923 (PMC13035797; doi:10.3389/fpls.2026.1771923)
Supplement: Supplementary file 4 [file Image4.pdf]

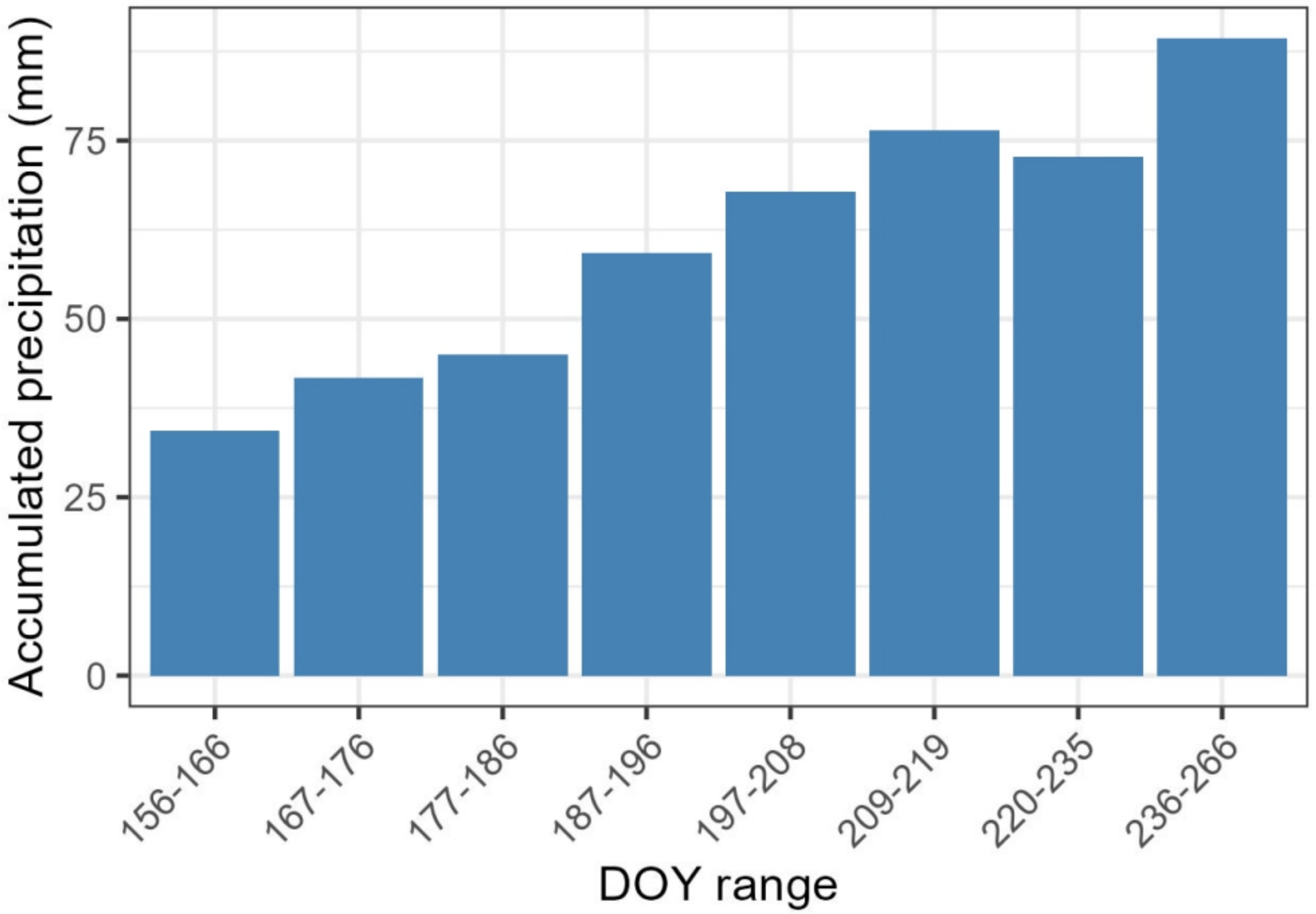

Figure S4 Accumulated precipitation within intervals across the growing season, registered with a rain gauge at the experimental site.
