# Supplementary material for: Insights into post-fire establishment of three Alpine conifer species after an experimental fire in Tyrol, Austria
Source: Front Plant Sci. 2026 Mar 17;17:1771923. doi: 10.3389/fpls.2026.1771923 (PMC13035797; doi:10.3389/fpls.2026.1771923)
Supplement: Supplementary file 5 [file Image5.pdf]

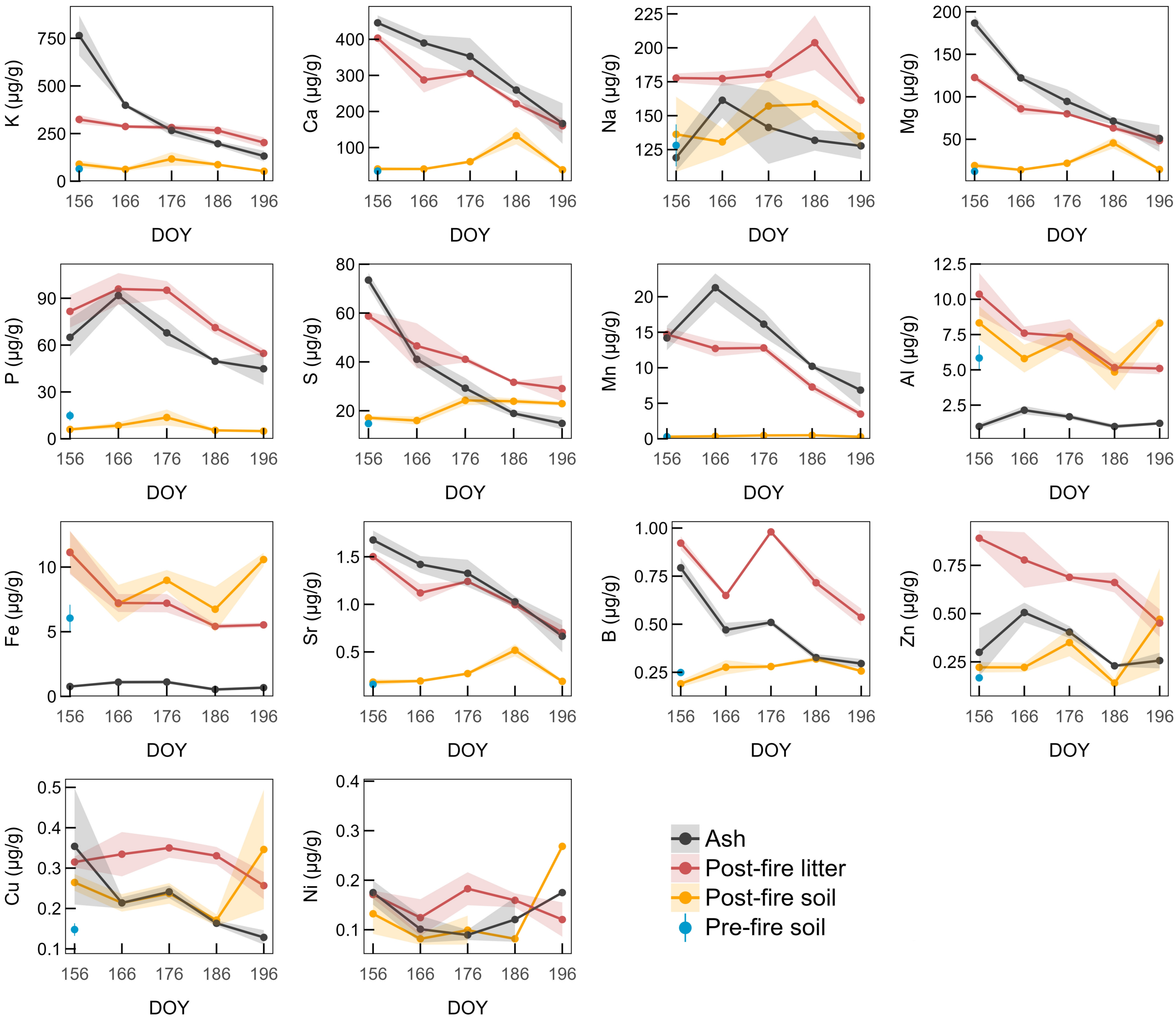

Figure S5 Concentrations of relevant elements in the fire plot. Measurements were taken from pre-and post-fire soil components, with components indicated by different colors. Pre-fire soil samples (blue) from the fire plot were analyzed on DOY 156. Elements with concentrations below detection limits are not presented. Data are means ± SE (shaded areas); triplicate measurement.
