# Supplementary material for: Insights into post-fire establishment of three Alpine conifer species after an experimental fire in Tyrol, Austria
Source: Front Plant Sci. 2026 Mar 17;17:1771923. doi: 10.3389/fpls.2026.1771923 (PMC13035797; doi:10.3389/fpls.2026.1771923)
Supplement: Supplementary file 6 [file Table1.docx]

Supplementary Material

Table S1 Reference climatic characteristics of the study site Paxmar (47° 09’N/11° 06’E, 1670 m a.s.l.). Climate data is represented by the nearest meteorological stations: Neustift-Mildes (47°06′ N/ 11°18′ E, 1030 m) and Patcherkofel (47°22′ N/ 11°47′ E, 2251 m). The values represent means calculated for the period January 2019 to January 2024. Air temperatures (indices D–G) were measured at 2 m above ground level. Data source: Geosphere (accessed 18 January 2026).

| Weather station | | Neustift Milders | Patscherkofel |
| --- | --- | --- | --- |
| Index | Climate period (Jan 2019 - Jan 2024) |  |  |
| A | Max daily precipitation (mm) | 56.8 | 45.0 |
| B | Annual precipitation (mm) | 996.0 | 928.3 |
| C | Relative humidity (%) | 77.4 | 76.2 |
| D | Mean annual T_max_ (°C) | 13.7 | 4.8 |
| E | Mean annual T_min_ (°C) | 3.6 | -0.8 |
| F | Absolute T_max_ (°C) | 31.8 | 21.4 |
| G | Absolute T_max_ (°C) | -13.4 | -17.4 |
| H | T_mean_ 0 -10 °C (days) | 143.8 | 153.5 |
| I | T_mean_ 10 - 20 °C (days) | 146.7 | 62.0 |
| J | T_mean_ 20 - 30 °C (days) | 21.7 | 0 |
| K | T_mean_ ≥ 30°C (days) | 0 | 0 |
| L | Precipitation 0.1 - 10 mm (days) | 143.2 | 147.0 |
| M | Precipitation 10 - 20 mm (days) | 18.8 | 20.2 |
| N | Precipitation 20 - 50 mm (days) | 9.0 | 7.8 |
| O | Precipitation ≥ 50 mm (days) | 0.7 | 0.3 |
