# Supplementary material for: Insights into post-fire establishment of three Alpine conifer species after an experimental fire in Tyrol, Austria
Source: Front Plant Sci. 2026 Mar 17;17:1771923. doi: 10.3389/fpls.2026.1771923 (PMC13035797; doi:10.3389/fpls.2026.1771923)
Supplement: Supplementary file 7 [file Table2.docx]

Supplementary Material

Table S2 P-values for the GAM model testing seedling establishment.

| Species | Sowing DOY |  | Control is significantly different from 0 | Fire is significantly different from 0 | Fire is significantly lower than control |
| --- | --- | --- | --- | --- | --- |
| *Pinus cembra* | 156 |  | 3.08E-14 | 0.000296 | 0.0000129 |
|  | 166 |  | 0.0377 | 0.255 | 0.488 |
|  | 176 |  | 0.00000153 | 1 | 0.000259 |
|  | 186 |  | 0.0000111 | 1 | 0.000847 |
|  | 196 |  | 0.000572 | 0.4387 | 0.0351 |
|  |  |  |  |  |  |
| *Picea abies* | 156 |  | 0.000000015 | 0.674 | 0.0000431 |
|  | 166 |  | 0.000696 | 0.0934 | 0.1779 |
|  | 176 |  | 0.0000382 | 0.0747 | 0.0518 |
|  | 186 |  | 0.00269 | 0.00594 | 0.829 |
|  | 196 |  | 0.00103 | 0.00789 | 0.03599 |
|  |  |  |  |  |  |
| *Larix decidua* | 156 |  | 0.000000032 | 0.701 | 0.0000654 |
|  | 166 |  | 4.99E-08 | 0.827 | 0.0000507 |
|  | 176 |  | 0.0000624 | 1 | 0.00288 |
|  | 186 |  | 0.000783 | 0.0105 | 0.483 |
|  | 196 |  | 0.0000605 | 0.3398 | 0.0111 |
